# Soft Tissue Sarcomas of the Arm – Oncosurgical and Reconstructive Principles within a Multimodal, Interdisciplinary Setting

**DOI:** 10.3389/fsurg.2016.00012

**Published:** 2016-02-23

**Authors:** Georgios Koulaxouzidis, Filip Simunovic, Holger Bannasch

**Affiliations:** ^1^Department of Plastic and Hand Surgery, University of Freiburg Medical Center, Freiburg, Germany; ^2^Section for ­Plastic-Reconstructive Tumor Surgery, University of Freiburg Medical Center, Freiburg, Germany

**Keywords:** soft tissue sarcoma, plastic-reconstructive surgery, upper extremity reconstruction

## Abstract

Soft tissue sarcomas of the upper extremity represent a severe threat for the patient and a difficult task for the treatment team. Due to the complex anatomy of the arm, most sarcomas involve valuable functional structures. Nonetheless, a large portion of the patients can be treated in a limb-sparing manner, and surgery is the mainstay of local tumor control. This review gives an overview of the disease entities and their epidemiology, on necessary patient work-up, staging, and imaging modalities, as well as the importance of interdisciplinary decision-making. The surgical therapies and principles of tumor excision are outlined, as well as reconstructive options. Furthermore, adjuvant treatments are discussed with a special focus on the various application techniques for radiation therapy. In spite of established treatment algorithms, each case is an individual challenge and individually tailored therapy is required. This aspect is illustrated by presenting three comprehensive cases demonstrating useful strategies. A summary of the relevant literature is given.

## Introduction

Soft tissue sarcomas (STS) are a heterogeneous group of rare malignant mesenchymal tumors representing <1% of newly diagnosed solid tumors a year (1). They have an annual incidence of two to three cases per 100,000 (2). Although sarcomas can occur throughout the body, 60% of STS in adults occur in the limbs (15% in the upper extremity and 45% in the lower extremity) (3). One-fifth of all STS occur in the upper extremity (4). Therefore, this condition is very rare, and consequently, the literature is limited to small case series (5–9). Compared to the lower extremity, where liposarcoma and myxoid sarcoma are commonly encountered; synovial sarcoma, epithelioid sarcoma, and fibrosarcoma are relatively more common in the upper extremity (10).

Treatment of upper extremity STS presents a major challenge as the complex anatomy and high functional demands compete with oncological safety demands. Until the 1970s, extremity STS often required limb amputation due to high rates of local recurrence (11, 12). Nowadays, limb-sparing surgery can be performed in more than 90% of the patients without compromise in local recurrence rates or survival rates (8, 13). This development reflects the success of a modern treatment concept with a multidisciplinary team approach (7, 14–18). However, surgical resection remains the cornerstone of treatment, and surgical resection margins are the main prognostic factor for local and systemic tumor control (6, 8, 19). In this review, we highlight key principles of oncological and reconstructive surgery of the upper extremity with focus on the upper arm and the forearm.

## Diagnostic and Staging Work-Up

Patients usually present with a subcutaneous tumor discovered by palpation. STS of the upper extremity are more readily noticed by the patient compared to the lower extremity. The estimation of duration and progression of the tumor is often uncertain, and there is no association to traumatic injury. The clinical behavior of STS is mostly misleading as they are characterized by slow growth, few physical complaints, and harmless appearance in cross-sectional imaging (20). However, due to the anatomic proximity of functional and neurovascular structures at the upper extremity complains of nerve involvement or functional impairment at first presentation are more common than in the lower extremity. Due to these circumstances, tumors of the upper extremity tend to be smaller at the time of presentation. In any case, every refractory swelling that is not recurrent after 4 weeks should initiate diagnostic work-up, as described and discussed in this article and elsewhere (21).

It is generally recommended that STS should be treated in tumor centers (16, 22–28). However, given the rarity and diversity of these tumors, it is not surprising that excisions are often performed without preoperative suspicion of malignancy and adequate preoperative diagnostic and staging work-up (22, 26, 29). Tumors of the upper extremity are twice as likely to undergo unplanned excision, probably because of their smaller size and more superficial location (30). In our own patient collectively, more than 50% of the patients are referred after such a procedure for further treatment. As shown, resection margins and histopathological assessments from referring institutions are often unreliable and unsuitable for further treatment planning. Moreover, especially in the upper extremity, the risk for residual tumor after unplanned primary excision is high. Although tumors are usually smaller than in the lower extremity, complex regional anatomy may be the reason for close margins or positive margins, necessitating more frequently postoperative radiotherapy. There is significantly more frequent residual disease and local recurrence in the upper extremity, especially around the elbow (10). Completion of diagnostic and staging work-up followed by re-excision is required in this patient group.

Contrast-enhanced MRI represents the standard procedure of diagnostic imaging, as it provides a detailed three-dimensional anatomic presentation of the tumor. With this information, tumor biopsy and excision can be planned (20, 31). A diagnosis can only be achieved by histopathological examination of a representative tumor sample. Therefore, excisional (only for small, superficial tumors) or incisional/punch biopsy has to be performed, as described elsewhere (21, 32). Second opinion reference-center pathology should be indicated generously.

Additionally, before a definitive treatment plan is established, completion of the clinical staging is mandatory. The assessment of tumor size, nodal status, and presence of metastasis has to be performed (TNM status) (33). A spiral CT of the thorax is indicated, as STS are primarily characterized by pulmonary metastasis. However, at the time of diagnosis, only 10% of the patients present with pulmonary metastasis (34). Only rarely, adult type STS metastasize to regional lymph nodes. Nevertheless, there are some sarcoma subtypes (synovial sarcoma, vascular sarcomas, rhabdomyosarcomas, and epithelioid and clear cell sarcomas) with a higher probability of lymphatic spread. However, the role of sentinel lymph node biopsy in these sarcoma subtypes is still unclear (35, 36). Although positron emission tomography (FDG–PET) has not been yet established as standard imaging modality, it has some relevant advantages. As a functional imaging procedure, FDG–PET provides, besides the metastatic situation of the whole body, data on tumor activity and treatment response after neoadjuvant treatment. However, limited experience, availability, and examination costs are actually restricting factors for standardized use (37).

## Tumor Board Review

Categorization of the patients takes place according to the American Joint Committee on Cancer/International Union against Cancer classification (33, 38). A thorough treatment plan is determined in an interdisciplinary tumor board setting, including oncosurgical, reconstructive, neoadjuvant, and adjuvant treatment options.

## Prognostic Factors

Complete tumor resection is the most important prognostic factor for local and distant disease control (8, 9). If local tumor control is not achieved, there can be no successful treatment. Similarly, the general patient outcome is strongly correlated to distant metastasis. In case of insufficient primary excision and evidence of residual tumor, maximum effort should be made to achieve complete resection by re-excision. Re-excision has to include all previous incisions and drainage exit sites as well as every anatomic compartment suspicious for contamination. Furthermore, the presentation status, whether it is a primary or recurrent tumor, as well as the tumor size (<5 or >5 cm), tumor grading, and extracompartimental location are relevant prognostic factors (8, 9). The specific anatomic tumor location is also a relevant factor: for example, tumor location at the shoulder girdle hampers limb-sparing surgery (39–41). As previously stated, residual disease and recurrence are more common in the upper extremity. Besides anatomical factors, this is also due to the fact that histological entities, which are more common in the upper extremity (angiosarcoma, malignant peripheral nerve sheath tumor), are associated with a higher risk of recurrence (42) that more frequent tumors of the lower extremity, such as low grade liposarcoma.

## Oncosurgical Resection

The extent of surgical margins is subject of discussion, as there is no solid evidence. The trend during the past decades has been directed toward reduction of the margins (43, 44). The most frequent type of excision is termed “wide excision,” signifying that the tumor is to be removed within healthy tissue, in the manner that the tumor is not seen by the surgeon. Contrastingly, the so-called “marginal excision” (excision at the level of the tumor capsule) is not adequate since it does not include a macroscopic layer of uninvolved tissue, which is required by the pathologist in order to certify an R0 status. Resection of the entire compartment, involving all muscles from their origin to the insertion is largely historical and is rarely required. Recently, the type of connective tissue involved was a topic of interest, as sarcomas grow along, rather than transverse, major fascial planes. This enables preservation of major nerves, bones, and muscles with <1 cm resection margin, when fascial planes can be included in the resection (45). Microsurgical techniques enable us to maintain the balance between radical resection and preservation of function. Tumor invasion of nerves is a rare occurrence. In case of close proximity to the tumor, epineural dissection is a safe option (46). Furthermore, neoadjuvant, intraoperative (IORT), or adjuvant radiotherapy treatment modalities additionally contributed to the reduction of resection margins. However, when neurovascular or functional structures cannot be preserved, the oncosurgical defects have to be incorporated into the reconstructive plan.

In cases of advanced disease and extensive infiltration, amputation of the limb is required. Also in these cases, there are useful reconstructive options, such as composite-tissue elbow transfer for reconstruction of the shoulder silhouette (41). In order to offer consultation to these patients, the surgeon also needs to be aware of the current advances in bionic prosthetics, which is a rapidly evolving field offering good solutions. The resected specimen should be clearly marked with sutures and the corresponding sites in the tumor bed with clips. An intraoperative biopsy examination is generally not recommended.

## Reconstruction

Whenever possible, reconstruction should be achieved as a one-step procedure during tumor excision. Otherwise, wound closure by vacuum-assisted closure can be an alternative, especially when resection margins are uncertain.

The goal of reconstruction is to provide reconstruction of every resected tissue and the related function and to achieve primary wound healing in the interest of timely rehabilitation and adjuvant therapy. In modern reconstructive surgery, it is not sufficient to merely achieve wound closure. It is just as important to strive for optimal functional and esthetic results. For example, split-thickness skin grafts can be transplanted on vital muscle tissue with success. However, this will not lead to an esthetically pleasing result, and it can possibly cause extensive wound healing problems, especially if tendons are exposed. In our patients, almost 70% of the patients with upper extremity sarcoma require reconstructive surgery, compared to 50% at the lower extremity. Reconstructive techniques are more often required in the distal parts of an extremity. In most of these patients, the whole reconstructive armamentarium has to be utilized (16, 18). Due to small case numbers, there is no evidence supporting the use of individual reconstructive techniques, so that the plastic surgeons use the traditional “reconstructive-ladder” when devising the treatment plan. It is, however, common for experienced surgeons, especially in the light of increasing expertise and confidence in free-flap surgery, to skip one or more ladders in the decision-making process.

Several authors have reported that local or regional flaps are associated with higher complication rates and have inferior functional results compared to free flaps (7, 47), which is also our own experience. Some authors found that free-flap reconstruction after soft tissue sarcoma excision at the upper extremity is associated with increased morbidity but better local control (48). Others found that functional outcome achieved satisfactory levels with both pedicled and free flaps (49).

Proximal upper extremity can be successfully treated with traditional random-pattern flaps or axial-pattern flaps originating from the extremity (lateral upper arm flap) or the shoulder (subscapular vascular territory). Axial flaps, such as dorsal interosseous flap, can be used on the forearm. Local flaps that compromise one of the major vessels, such as the pedicled radial artery flap, are not recommended any more due to extensive donor-site morbidity.

There are several free-flap options with minimal donor-site issues, which are used routinely. Since there is no advantage in using a muscle flap and since there are several good perforator flaps available, the perforator flaps have become the mainstay of modern reconstructive surgery. The workhorse flap for extremity reconstruction is the anterolateral thigh (ALT) flap (17), which enables a two-team parallel approach in the supine position. The parascapular flap is another good alternative, requiring surgery in the lateral position. The thoracodorsal artery perforator (TDAP) flap or the musculocutaneous latissimus-dorsi (LD) flap is available for larger defects.

Functional reconstruction of nerve defects (commonly using sural nerve grafting) or blood vessel interposition is also performed at this stage. If there is a tendon defect and the muscle can be preserved, free tendon transplants are performed, usually using the palmaris longus or plantaris tendon. If the muscle has to be sacrificed, the first-line solutions available are the classical tendon transfers, e.g., radial nerve palsy tendon transfer (50). Functional free flaps are rarely required. In this case, free functional gracilis transfer is the primary option, e.g., for reconstruction of finger flexion, as shown in case 3.

## Adjuvant Therapy

### Radiation Therapy

Radiation therapy is almost always used as neoadjuvant therapy. The only indication for radiation monotherapy is rare cases that are deemed inoperable because of significant comorbidities. Using the combination of surgical and radiation therapy, 90–95% rates of limb salvage can be achieved. Radiation is usually administered as adjuvant therapy, in a combined dosage of 60–66 Gy with conventional fractionation. The field of radiation includes the tumor bed, margin of safety, as well as scars from previous operations and drainage exit sites. In this way, an improvement of local control for G2 and G3 STS can be achieved. Radiation for G1 tumors is not indicated after R0 resection. Adjuvant radiation can negatively influence complex reconstruction, so that high-grade sarcoma requiring extensive surgery should preferably be treated with neoadjuvant radiation (51). Neoadjuvant radiation has comparable effects on local disease control as adjuvant therapy. The advantages are that the field of radiation can be kept smaller, and the dosage required (50 Gy) is lower. The often discussed severe postoperative wound healing complications after neoadjuvant radiotherapy are much more frequent in the lower extremity (52). Thus in the upper extremity, this strategy can be applied more liberally by taking advantage of the smaller irradiation field and dose. Intraoperative radiation therapy is always used in addition to neoadjuvant radiation therapy, the dosage of which can be reduced accordingly. Usually, 12–15 Gy are administered to the tumor bed (case 2).

### Chemotherapy

Most types of STS are not very sensitive to chemotherapy, with exception of small-round-blue-cell tumors, extraosseous Ewing sarcoma, rhabdomyosarcoma, primitive neuroectodermal tumor, and desmoplastic small-round-cell tumor. Despite an increase in experimental data, there is still limited application of molecular-targeted therapy for treatment of STS. Two examples of successful targeted therapy are the use of imatinib in treatment of dermatofibrosarcoma protuberans and the use of sorafenib in treatment of angiosarcoma. The use of standard chemotherapeutics, such as anthracyclines or ifosfamide after R0 resection, is not generally recommended.

### Hyperthermia and Isolated Limb Perfusion

The combination of neoadjuvant or adjuvant chemotherapy and regional hyperthermia, possibly with additional radiation therapy, can improve local disease control in locally advanced tumors. Isolated limb perfusion with tumor necrosis factor alpha or melphalan can be considered in tumors, which cannot be resected with R0 margins, or when surgical excision would lead to mutilating loss of function.

## Conclusion

To achieve optimal outcomes, treatment of STS of the upper extremity should be carried out at experienced institutions where all relevant disciplines are represented. Each sarcoma has its specific histopathological phenotype, grading, size, and, importantly, specific anatomical localization within the complex anatomy of the arm.

Nevertheless, widely accepted treatment principles exist that can be adapted to the individual situation. Prior to treatment, it is mandatory to discuss the case in a multidisciplinary conference defining the use and the sequence of treatment modalities. The mainstay of local tumor control – the prerequisite for curing the patient – is surgery. A radical oncosurgical approach in the upper extremity requires plastic-reconstructive procedures in more than 70% of the cases. The continuously expanding plastic-surgical options for reconstruction of surface, volume, and function enable surgeons to customize the oncosurgical procedure and to preserve the upper extremity with a good oncological and functional result in most of the cases.

## Case Demonstrations

**Figure 1:**
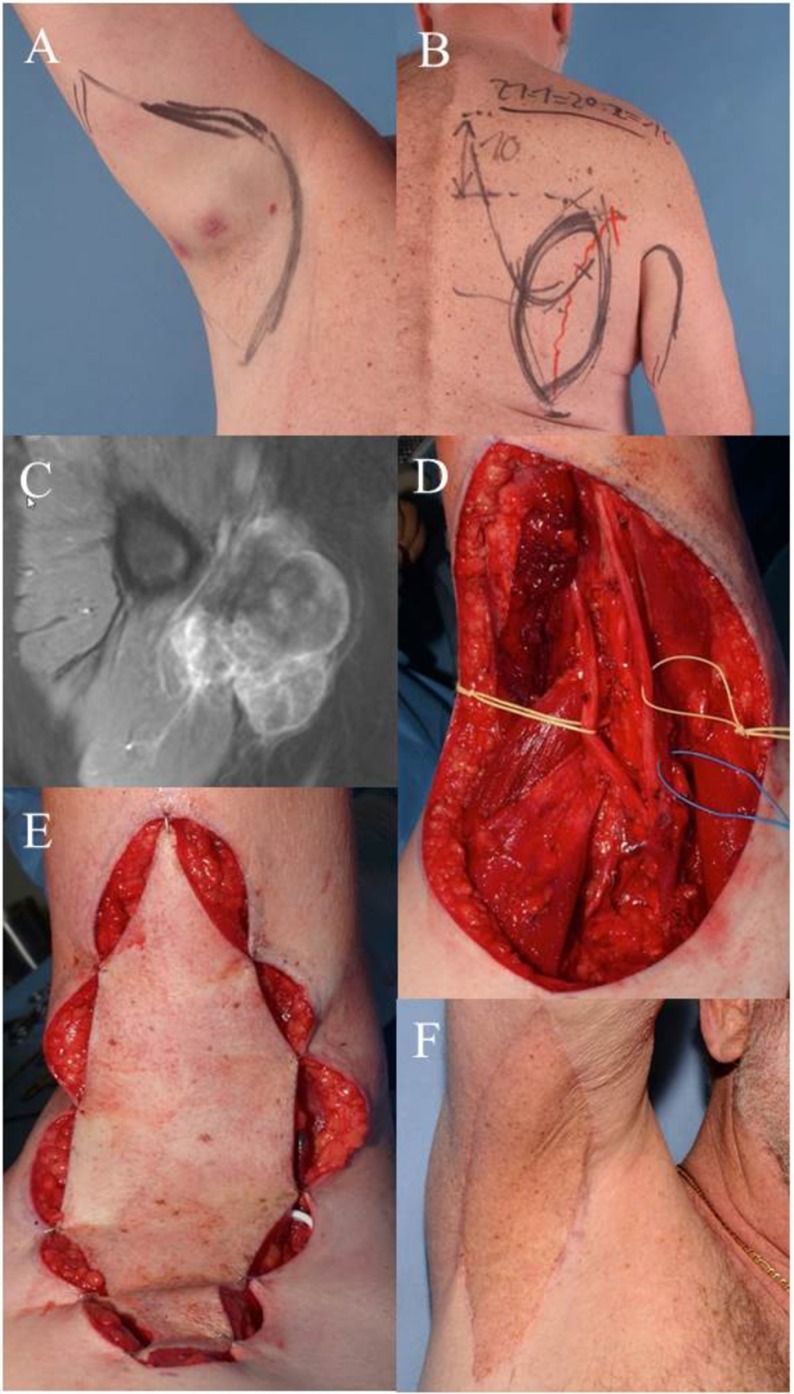
**Case 1: (A) clinical visible mass in the right axilla, pre-OP drawing of the wide resection margins**. **(B)** Pre-OP drawing demonstrates planned pedicled parascapular fasciocutaneous flap. **(C)** Pre-OP MRI of the right axilla. **(D)** Intraoperative situation after wide en-bloc excision of tumor. Deep margins were achieved by epineurectomy and adventitiectomy. **(E)** Pedicled parascapular flap inset reconstructs the defect. **(F)** Clinical appearance 5 years after surgery and external post-OP radiation therapy.

Case 1 (Figure 1) demonstrates the case of a 64-year-old male patient suffering from a high-grade pleomorphic sarcoma in the right axilla (T2b, N0, M0, G3). A wide excision was planned and carried out with sufficient removal of skin and subcutaneous tissue due to the extracompartimental localization of the sarcoma. Margins to the deep structures of the axilla could be achieved by thorough dissection of the nerve and vessel sheaths. The transfer of a pedicled parascapular flap was performed in the same operation. Noteworthy, we dissect the pedicle completely back through the medial axillary gap, ligating the osseous branches of the circumflex scapular artery. The flap can be advanced through this tunnel, allowing a completely tension-free placement in the axilla. The patient is shown 5 years after surgery and post-OP radiation therapy with an excellent functional result. The parascapular flap delivers sufficient pliable soft tissue coverage avoiding functional impairment of shoulder movement (53–55).

**Figure 2:**
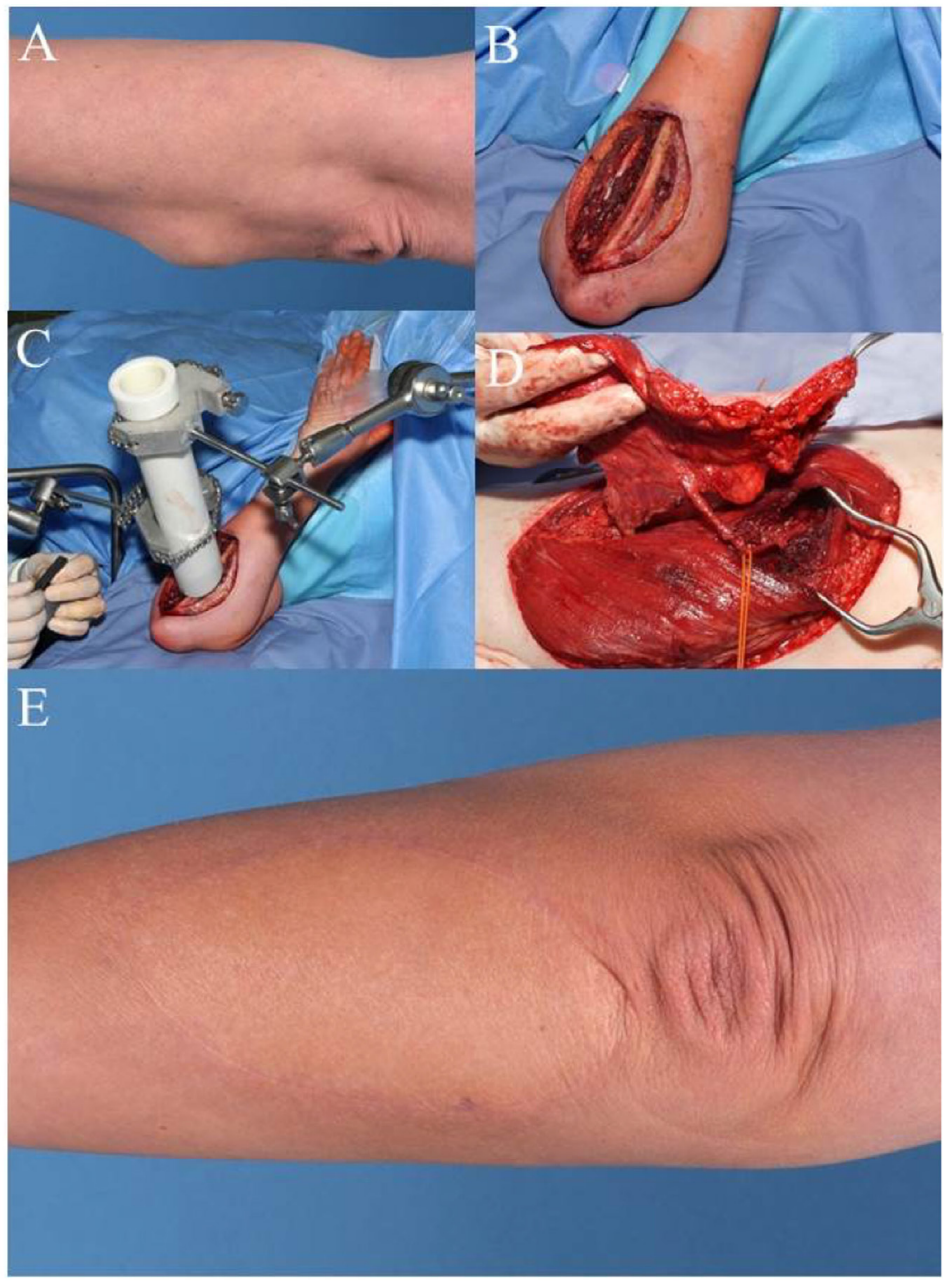
**Case 2: (A) pre-OP picture shows mass in the proximal forearm**. **(B)** Intra-OP situation after wide en-bloc excision. **(C)** Intra-OP application of radiation therapy in the wound bed close to the ulna (15 Gy). **(D)** Raised anterolateral thigh flap from the left leg for microvascular reconstruction. **(E)** Clinical appearance of the reconstructed forearm 1 year after additional external radiation therapy (50.4 Gy).

Case 2 (Figure 2) shows a 47-year-old female patient with fast recurrence of an incompletely resected high-grade sarcoma of the left forearm (pleomorphic sarcoma, initially T1b, N0, M0, G3). She had underwent the so-called “whoops procedure” – a term referring to an unplanned sarcoma resection where no malignancy had been suspected. A wide resection was performed with partial removal of the ulnar periosteum. The deep tumor bed was treated with an internal radiation therapy with the application of 15 Gy, thus reducing the post-OP dose from 65 to 50 Gy. Distal to the elbow, large defects are difficult to cover with local tissue transfer. The transfer of a free-flap offers an elegant option to avoid additional morbidity in proximity to the tumor region. The demonstrated ALT flap is one of the “workhorse” microvascular flaps in reconstructive tumor surgery of the extremities (17).

**Figure 3:**
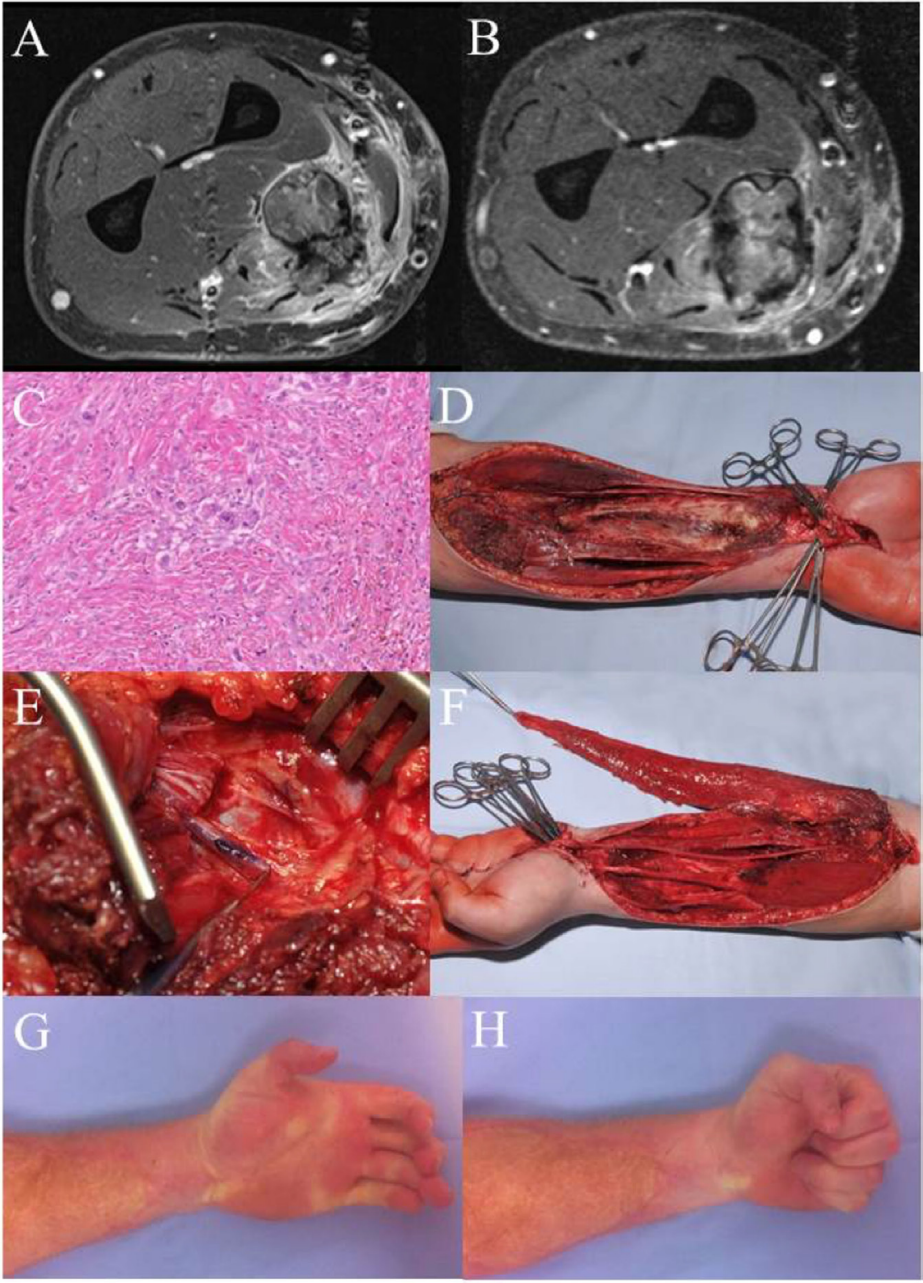
**Case 3: (A) MRI left forearm before radiation**. **(B)** MRI left forearm post radiation. **(C)** H&E specimen showing only few scattered remaining tumor cells as a result of the preoperative radiotherapy. **(D)** Intraoperative situation after en-bloc excision of the flexor compartment. **(E)** Intraoperative detail photography of the transected median nerve. The blue marking shows the motor branch to the deep flexors, later used for nerve coaptation to the obturator branch of the transferred gracilis muscle. **(F)** Gracilis muscle after microvascular transfer and motor nerve coaptation. After defining the proper tension, the muscle will be fixed to the deep flexor tendons (II–V). Flexor pollicis longus function was reconstructed using a brachioradial tendon transfer. **(G,H)** Clinical result 5 years after therapy. There is a remaining extension deficit, but a full finger flexion with a strong grip could be achieved. Patient is in complete remission and rehabilitated in his original job as a truck driver.

Case 3 (Figure 3) shows a 29-year-old patient with an epithelioid cell sarcoma (T2b, N0, M0, G3) of the left forearm within the flexor compartment. Crucial structures (flexor muscles, median nerve, and radial artery) are involved. When complex functional reconstruction of those structures is necessary, postoperative radiation therapy should be avoided, which is why preoperative radiation therapy (50 Gy) was conducted in this case. The MRI pre- and postradiation do not differ much in tumor size, but a significant reduced contrast enhancement can be demonstrated. Histology reflects good response to radiation therapy with only few vital, scattered tumor cells. Radical resection was carried out with en-bloc removal of all flexors (except FCU), the median nerve, and the radial artery. Sensory reconstruction was performed *via* multiple sural nerve cable grafts, which resulted in recovery of protection sensation. Functional reconstruction for the flexors was achieved with a free microvascular, functional gracilis muscle transfer, covered with a split-thickness skin graft (56). The patient has recovered quite well from this radical approach and is rehabilitated in his former job as truck driver. In the upper extremity, much less wound healing complications arise from this sequence of treatment modalities, especially when free tissue transfer is performed. In the lower extremity, there is a higher occurrence of severe wound healing complications after preoperative radiation.

## Ethics Statement

Written informed consent was obtained from the patients prior to presenting the cases.

## Author Contributions

GK: data acquisition, literature search, manuscript preparation, and final approval. FS: data acquisition, critical manuscript review, literature search, and final approval. HB: data acquisition, case reports, critical manuscript review, and final approval.

## Conflict of Interest Statement

There is no conflict of interest. There was no payment or services from a third party for any aspect of the submitted work.
